# Theoretical Investigation of a Highly Sensitive Refractive-Index Sensor Based on TM_0_ Waveguide Mode Resonance Excited in an Asymmetric Metal-Cladding Dielectric Waveguide Structure

**DOI:** 10.3390/s19051187

**Published:** 2019-03-08

**Authors:** Xiangxian Wang, Xiaoxiong Wu, Jiankai Zhu, Zhiyuan Pang, Hua Yang, Yunping Qi

**Affiliations:** 1 School of Science, Lanzhou University of Technology, Lanzhou 730050, China; wxxwgrlz1609@163.com (X.W.); zhujk314@163.com (J.Z.); pangzhiyuan429@126.com (Z.P.); hyang@lut.cn (H.Y.); 2 College of Physics and Electronic Engineering, Northwest Normal University, Lanzhou 730070, China; yunpqi@126.com

**Keywords:** refractive index sensor, waveguide mode, sensitivity, resonance wavelength, figure of merit

## Abstract

This study proposes a highly sensitive refractive-index (RI) sensor based on a TM_0_ waveguide mode resonance excited in an asymmetric metal-cladding dielectric waveguide structure, where the analyte serves as the guiding layer. By scanning the wavelength at fixed angles of incidence, the reflection spectra of the sensor were obtained. The results showed that the resonance wavelength redshifted dramatically with increases in the analyte RI, which indicates that this approach can be used to sense both the resonance wavelength and the analyte RI. Based on this approach, we investigated the sensing properties, including the sensitivity and figure of merit, at fixed incident angles of 60° and 45°, at which the sensitivity of the sensor reached 7724.9 nm/RIU (refractive index units) and 1339 nm/RIU, respectively. Compared with surface plasmon resonance sensors, which are based on a similar structure, the proposed sensor can accept a more flexible range of incident angles and a wider sensing range of analyte RI. This approach thus has tremendous potential for use in numerous sensing domains, such as biochemical and medical analyses.

## 1. Introduction

Surface plasmon resonance (SPR) can generate a strong electromagnetic field enhancement on the surface of a metal structure, and is very sensitive to the surrounding environment. Therefore, SPR can be applied to numerous fields such as absorption enhancement [1,2], magnetic field enhancement [3,4], photocatalysis [5,6,7,8,9,10], THz oscillation [11,12,13], Fano resonance [14,15,16,17], surface-enhanced Raman scattering (SERS) [18,19,20,21], sub-wavelength lithography [22,23], and refractive index (RI) sensors [24,25,26,27,28,29]. For refractive index sensors based on SPR, the resonance is excited mainly in two modes, the angular mode (at fixed wavelength) and the spectral mode (at fixed angle) [30,31]. Furthermore, among the variety of structures used to excite SPR, two main structures are used in biochemical sensors that are based on prism- or grating-coupling structures [30,31]. Recently, many studies have reported results dealing with these sensing mechanisms. For instance, Byun et al. designed a grating-coupled transmission-type SPR sensor by optimizing the structure of dielectric and metallic gratings on a metal film and obtained a sensitivity of 69.571 degrees/RIU (refractive index units) using angle scanning [32]. In addition, Abutoama et al. realized a sensitivity of 580 nm/RIU using a self-referenced dielectric grating structure [30]. However, using the grating-coupled method, this sensor led to a lower sensitivity when using wavelength interrogation [33], and had higher fabrication costs due to the added complexity of the gratings [34]. In contrast, prism-based structures for SPR sensing offer relatively obvious advantages, such as a higher sensitivity and simpler operating flexibility [33]. Homola et al. calculated the sensitivity of a prism-based sensor system using the Fresnel equations and found that it peaked at 970 nm/RIU, which was over three times the sensitivity of grating-based sensors under the same conditions [35]. Meanwhile, Cahill et al. proposed a much better sensor the highest sensitivity of which was able to reach approximately 1.681 × 10^−4^ RI units at a resolution of 1 nm, which was equivalent to 5948 nm/RIU [36]. Nevertheless, when using sensors that rely on a prism-coupled SPR method, the analyte’s RI must be less than that of the prism, which limits its applicability in some cases [37].

In contrast to the prism-coupled SPR sensing method, we previously explored an improved RI sensing technique based on an asymmetric metal-cladding dielectric waveguide (AMDW) [34,38,39]. Based on this AMDW structure, we had considered a method to fabricate hierarchical, sub-wavelength, photonic structures with various periods and number of layers via high-order waveguide-mode interference [34]. Furthermore, we also presented a fabrication method for complex, 2D, subwavelength structures by rotating and exposing the samples and using zeroth-order waveguide-mode interference [38]. Through these works, we found that the waveguide layer is extraordinarily sensitive to the RI of the dielectric. Thus, we initially considered angular scanning sensors and found a high sensitivity based on this AMDW structure [39]. It is noted that the detected analyte RI in the AMDW structure can even exceed that for prism-based methods. Nevertheless, this angular sensing method with a fixed incident wavelength requires mechanical rotation to continuously change incident angles, which is a complex operation with low efficiency. Thus, in this study we investigated the wavelength-scanning method using a broadband light source that simultaneously irradiated the analyte with different wavelengths, which showed obvious improvements over the angular-based RI sensing method.

Operating the wavelength scanning at a fixed angle, the reflectance spectrum can be detected using a spectrometer, and the close relationship between the analyte RI and the position of the wavelength resonance can be exploited to determine the RI. Therefore, this work used the AMDW structure to determine the RI while the sensing features were investigated based on a finite-element simulation (COMSOL multi-physics). Firstly, we calculated the reflectance spectrum given the refractive indexes of the analyte and the sensing properties for a 60° angle of incidence. Next, we simulated the optical field distributions of the AMDW resonances, which reflected the transfer of energy. Finally, to demonstrate the superiority of the proposed approach, we compared these results with those for a 45° angle of incidence and an SPR sensor. The results demonstrated the performance of the AMDW structure to sense the RI of the analytes.

## 2. Sensor Design and Analysis

**Figure 1:**
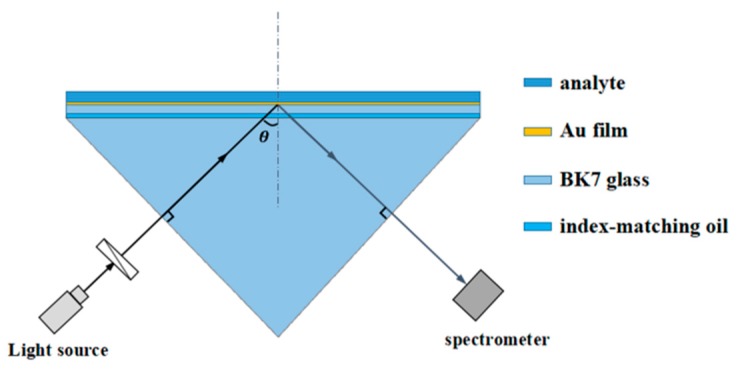
Schematic of the RI sensor based on the AMDW structure.

Figure 1 shows a schematic of the proposed sensor based on the AMDW structure. The design is similar to that described in Reference [39]. The substrate is BK7 glass, which is also used for the prism that couples into the waveguide mode resonance, and their RIs are set to be $n_{g}$. The analyte RI is denoted as $n_{a}$. Between the glass substrate and the prism, there is a layer of index-matching oil, so the three pieces can be considered as a single optical entity. The metal layer is an Au film with a $d_{Au}$ of 50 nm sputtered onto the glass substrate with its RI of $n_{Au}$. On top of the Au film, the analyte serves as a guided-wave layer with a $d_{a}$ of 500 nm. In addition, the infinitely thick air with an RI of $n_{air}$ serves as the cladding around the analyte, which is termed the dielectric cladding layer. Therefore, the sensor constitutes an ideal three-layer waveguide structure (Au film, analyte, and infinitely thick air). The incoming TM-polarized light irradiates one side of the prism at an incidence angle θ, following which the light transmits the polarization from the source, and the spectrometer accepts the refracted light on the other side of the prism. This structural system formed the basis for the proposed high-sensitivity, wavelength-scanning RI sensor at a fixed incident angle. The two sensing structures based on the angular-scanning and wavelength-scanning methods were similar; however, due to the low efficiency of continuously changing the incident angle in a real system, the wavelength-scanning method was preferred as a means for more rapid measurement of the reflection spectrum.

When the TM-polarized incident light satisfies certain conditions, the corresponding waveguide mode resonances occur within the AMDW structure. As this is an ideal three-layer waveguide, the eigenmode equation for the AMDW structure described in [39,40,41,42] is specified as follows.

(1) $$\kappa_{a}d_{a}=\;\mathrm{arc}tan\left(\alpha_{air}/\kappa_{a}\right)+\;\mathrm{arc}tan\left(\alpha_{Au}/\kappa_{a}\right)$$

where $\kappa_{a}$, $\alpha_{air}$ and $\alpha_{Au}$ are

(2) $$\{\begin{array}{c} \kappa_{a}=\sqrt{k_{0}^{2}n_{a}^{2}-\beta^{2}}\\ \alpha_{Au}\;=\;\sqrt{\beta^{2}-k_{0}^{2}n_{Au}^{2}}\\ \alpha_{air}\;=\;\sqrt{\beta^{2}-k_{0}^{2}n_{air}^{2}} \end{array}$$

with $k_{0}=2\pi /\lambda_{0}$ being the wave vector for the corresponding resonant vacuum wavelength $\lambda_{0}$. The propagation constant β satisfies

(3) $$\beta {\;=\;k}_{0}n_{g}sin\theta_{0}$$

where $\theta_{0}$ is the angle of incidence when the TM_0_ waveguide mode is resonant.

## 3. Sensor Performance

The finite element method (FEM) with periodic boundary conditions (in the horizontal direction) and perfectly matched layers (in the air) was utilized to simulate the reflection spectra and optical field distributions in the AMDW structure. In the simulation, the TM_0_ mode was excited by prism coupling, and the reflectivity was defined as *I_out_/I_in_*, where *I_in_* denotes the total intensity of light radiated from one side of the prism and *I_out_* denotes the total intensity of light emitted from the other side of the prism after being reflected by the AMDW. The reflection spectrum of our RI sensor was obtained by parametric wavelength scanning with a step of 1 nm. Furthermore, based on the above analyses and the simulated reflection spectra, we calculated the relationship between the resonance wavelength and the RI of the analyte. Moreover, the sensitivity and figure of merit (FOM) were calculated to depict the sensing performance.

**Figure 2:**
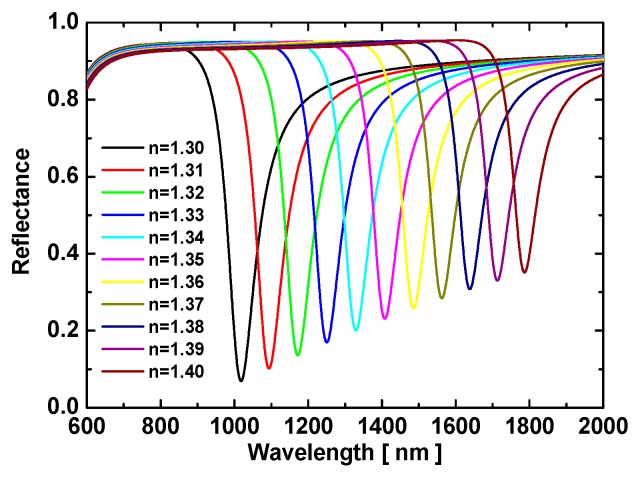
Reflection spectra of the AMDW structure for various refractive indices and for a fixed angle of incidence of 60°.

The two common prism types used in these devices are the equilateral-triangle prism and the isosceles right-triangle prism, which we used as coupling devices for sensing. Thus, the incident light had a natural overlap with its normal direction, which allowed a broad range of wavelengths to irradiate the metal film with consistent angles of incidence, which were set to 60° and 45°. Here, the reflection spectra were calculated when the light was normally incident on the air–prism interface and entered the prism to irradiate the Au film at 60°. Figure 2 shows the resulting reflection spectra using this configuration for various analyte RI, which were increased in steps of 0.01.

The results shown in Figure 2 clearly indicate that the TM_0_ resonance redshifted gradually and the dips rose with the increasing analyte RI. To further characterize the sensing capability of this scheme, we calculated the properties of this reflection dip.

**Figure 3:**
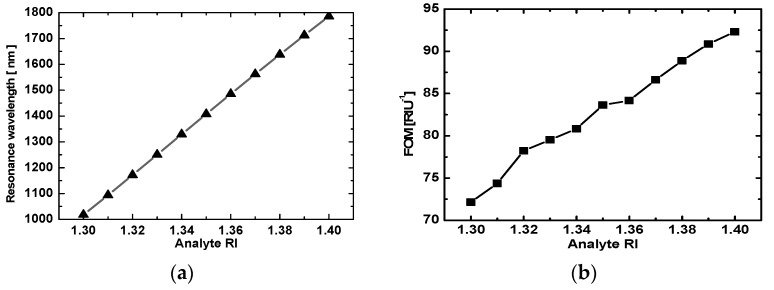
Two RI sensor properties for coupling via an equilateral triangular prism. (**a**) The resonance wavelength as a function of analyte RI with a linear fit produced a slope of 7724.9 nm/RIU; and (**b**) the FOM as a function of analyte RI.

The resonance wavelengths for these nanostructures were determined based on the analyte RI and two aspects of the nanostructure properties were used to evaluate the quality of an RI sensor, namely, the sensitivity and the FOM [43,44], which were defined as

(4) $$s=\frac{\Delta \lambda }{\Delta n}$$

(5) $$FOM=\frac{s}{FWHM}$$

where *Δλ* is the shift in the resonance wavelength relative to changes in the RI *Δn*, and the FOM is the ratio of the wavelength sensitivity to the resonance bandwidth, where “FWHM” stands for “full width at half maximum” [32]. Figure 3a plots the position of the TM_0_ resonance dip as a function of analyte RI, showing that the two are linearly related. Based on Equation (4), the slope of 7724.9 nm/RIU for the line in Figure 3a was the sensitivity obtained from wavelength scanning at a fixed 60° angle of incidence when using the equilateral prism for coupling. This sensitivity was much greater than previous reports [30,35,36], which attests to the remarkable performance of the proposed setup. In addition, the FOM was calculated using Equation (5) and plotted as a function of the analyte RI in Figure 3b. These two figures clearly show that the resonance wavelength and FOM increased with the increasing analyte RI. The FOM remained above 70 RIU^−1^ and reached a highest point of 92.3 RIU^−1^. Thus, the proposed method to determine the RI based on the resonance of the TM_0_ waveguide mode is clearly superior to previous techniques.

**Figure 4:**
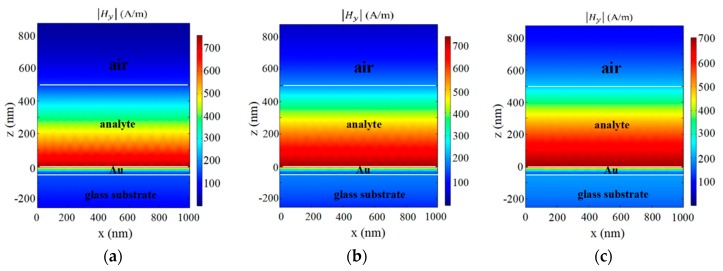
Simulated optical field distributions for three TM_0_ resonance conditions: analyte RI of (**a**) 1.30, (**b**) 1.35, and (**c**) 1.40.

Taking the upper surface of the metal film as the *x-y* plane, which was also the lower surface of the analyte, we further investigated the optical field distribution through numeric simulations. Based on the results of the simulated wavelength scan from 600 to 2000 nm, most of the energy was reflected from the glass–Au interface, which supported the reflections shown in Figure 2. However, the field was found to be mostly concentrated in the analyte layer when all the conditions were satisfied for a TM_0_ resonance dip. In other words, the TM_0_ waveguide mode resonance was excited when the parameters satisfied the equations of the AMDW structure. Figure 4 shows three simulated optical distributions for the resonance dips. These plots illustrate that the energy from the TM-polarized plane-wave light was transferred due to the interactions between the Au film and the analyte layer [45].

**Figure 5:**
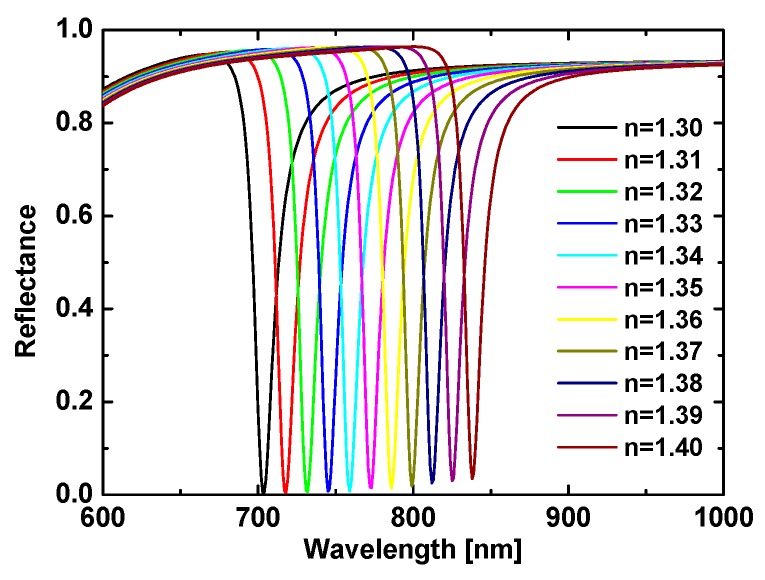
TM reflection spectra for AMDW structure and for several analyte RIs, all with a 45° angle of incidence.

**Figure 6:**
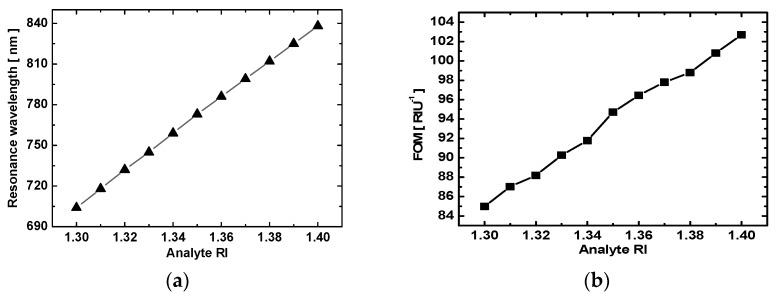
Sensing properties for the isosceles right-angle prism. (**a**) Resonance wavelength as a function of the analyte RI with a linear fit producing a slope of 1339 nm/RIU, and the (**b**) FOM as a function of the analyte RI.

In addition, we calculated the reflection spectra for a 45° angle of incidence when using an isosceles right-angle prism (see Figure 5). The resonance wavelength and FOM were plotted as functions of the analyte RI in Figure 6, as was done for the equilateral prism. Figure 5 shows that the resonance dip was narrower and attained a lower reflectivity at resonance than for the equilateral prism (see Figure 2). That is, the FOM was larger because the narrower FWHM outweighed the smaller sensitivity. Moreover, the resonance positions also changed. For the isosceles prism, the resonance wavelengths spanned from 704 to 838 nm; in contrast, they spanned from 1018 to 1786 nm for the equilateral prism.

To further clarify the sensing characteristics, Figure 6a,b plots the resonance wavelength and FOM for the isosceles prism. The resonance wavelength increased linearly with the analyte RI, giving a sensitivity of 1339 nm/RIU, which was distinctly smaller than for the equilateral prism, but the FOM was greater at the same analyte RI. Thus, each prism type offered its own advantages, allowing the selection of a broadband light source and angle of incidence to match the given circumstances. Although these results concern two selected angles of incidence (60° and 45°), similar sensors with different angles of incidence should have analogous properties.

**Figure 7:**
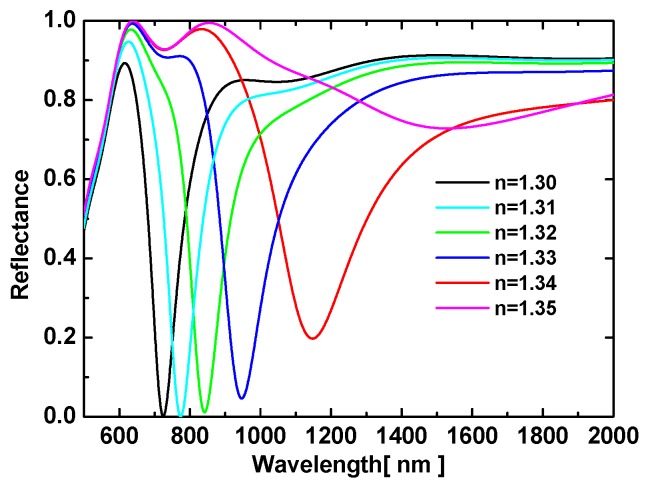
Reflection spectra for an SPR structure with an infinite analyte thickness.

**Figure 8:**
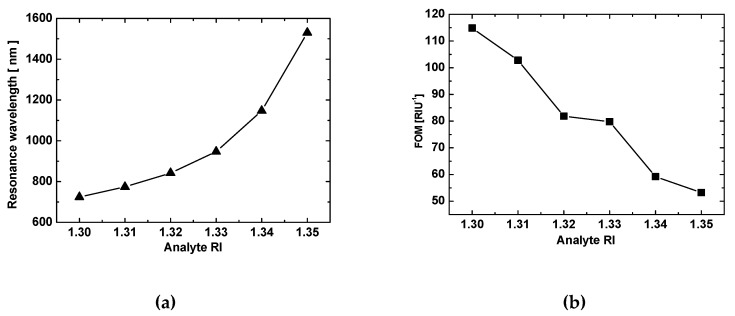
Resonance wavelength (**a**) and FOM (**b**) as a function of analyte RI for the SPR sensing structure.

Furthermore, to illustrate the advantages of the proposed sensors, we also calculate the SPR sensing properties for a similar structure that used an infinitely thick analyte instead of a 500-nm-thick layer. For angles of incidence of 60° and 45°, the SPR reflectivity spectrum only appeared to be better suited to determine the analyte RI when it was much less than the prism RI. In other words, the spectrum only had deep and narrow dips when this relationship was satisfied. Thus, after multiple attempts, 65° was chosen as the angle of incidence. Figure 7 shows the reflectance as a function of incident wavelength for several analyte RIs. The analyte RI was required to be much lower than the RI of the prism [37]; accordingly, this setup can only be used to determine the analyte RIs from 1.30 to 1.35 because, as is shown in Figure 7, the resonance dip vanishes when the analyte RI exceeds ~1.35. In addition, Figure 8a,b shows its resonance wavelength and FOM for the SPR sensing structure, and illustrates the resonance wavelength increases nonlinearly with the analyte RI while the FOM decreases rapidly despite the increasing sensitivity. Thus, the sensing method based on SPR seems only to be applicable over a small range of analyte RI and is not suitable for detecting RIs that exceed ~1.35.

## 4. Conclusions

In summary, we proposed a highly sensitive RI sensor that exploits the TM_0_ waveguide mode excited in an asymmetric metal-cladding dielectric waveguide structure. For various incident wavelengths and at the angles of incidence of 60° and 45°, the sensitivity of the setup was found to be as high as 7724.9 nm/RIU and had a reasonable FOM with a 60° angle of incidence for analyte RIs between 1.30 and 1.40. This was much greater than the results that were found for the 45° angle of incidence. These results also demonstrated that the capabilities of the sensor depended on the wavelength range of the TM_0_ waveguide mode resonances and the angle of incidence. This means that the light source and angle of incidence can be flexibly selected according to the requirements of the specific application. Compared with a similarly structured SPR sensor, the sensitivity of the proposed sensor was more stable and offered a better FOM, making it more effective for practical sensing measurements. The proposed method additionally enables the determination of the analyte RI over a wider range, which makes it more valuable for broader RI detection.
